# Oral Health’s Transformative Impact on Diet and Systemic Health Outcomes

**DOI:** 10.1093/geroni/igab046.965

**Published:** 2021-12-17

**Authors:** Michèle Saunders

## Abstract

The 2020-25 Dietary Guidelines for Americans identified dental caries as a major diet-related chronic disease of public health concern and suggested in the section for adults over 60, “Good dental health is critical to overall health, as well as the ability to chew foods properly." Poor oral health can lead to chronic diseases and impede one’s ability to chew fruits, vegetables, whole grains, and other nutrient-rich foods across the life span. Almost 90 percent of adults ages 20 to 64 years and 96 percent of those over 65 years of age have dental caries. The overall prevalence of complete tooth loss is 2.2 percent among adults ages 20 to 64 years and jumps to 17.3 percent for those over age 65. As a result of COVID-19, some seniors are not seeking regular oral health services, which increases the need for preventive oral health practices and consuming a healthy dietary pattern recommended in the new Dietary Guidelines. Recent research will underscore the importance of saliva and oral health in cancer patients on radiation and in other chronic diseases. Saliva has also been shown to reduce specific infections that are related to influenza and HIV. Participants in this session will gain understanding of factors linking poor oral health and nutrition practices to chronic diseases and guidance on critical preventive oral health practices to increase saliva flow and decrease dental caries through all stages of the life cycle. Promoting oral health is the responsibility of the interdisciplinary team overseeing older adults.

